# An improved nomogram including elastography for the prediction of non-sentinel lymph node metastasis in breast cancer patients with 1 or 2 sentinel lymph node metastases

**DOI:** 10.3389/fonc.2023.1196592

**Published:** 2023-06-05

**Authors:** Hongtao Duan, Jiawei Zhang, Guanxin Zhang, Xingmeng Zhu, Wenjia Wang

**Affiliations:** ^1^ Department of Ultrasound, Wuxi Huishan District People’s Hospital, Wuxi, Jiangsu, China; ^2^ Department of Ultrasound, Hulunbuir People’s Hospital, Hulunbuir, China

**Keywords:** nomogram, elastography, non-sentinel lymph node metastasis, breast cancer, axillary lymph node dissection

## Abstract

**Background:**

The rate of breast-conserving surgery is very low in China, compared with that in developed countries; most breast cancer patients receive mastectomy. It is great important to explore the possibility of omitting axillary lymph node dissection (ALND) in early-stage breast cancer patients with 1 or 2 positive sentinel lymph nodes (SLNs) in China. The aim of this study was to develop a nomogram based on elastography for the prediction of the risk of non-SLN (NSLN) metastasis in early-stage breast cancer patients with 1 or 2 positive SLNs.

**Methods:**

A total of 601 breast cancer patients were initially recruited. According to the inclusion and exclusion criteria, 118 early-stage breast cancer patients with 1 or 2 positive SLNs were finally enrolled and were assigned to the training cohort (n=82) and the validation cohort (n=36), respectively. In the training cohort, the independent predictors were screened by logistic regression analysis and then were used to conducted the nomogram for the prediction of NSLN metastasis in early-stage breast cancer patients with 1 or 2 positive SLNs. The calibration curves, concordance index (C-index), the area under the receiver operating characteristic (ROC) curve (AUC), and Decision curve analysis (DCA) were used to verified the performance of the nomogram.

**Results:**

The multivariable analysis showed that the enrolled patients with positive HER2 expression (OR=6.179, P=0.013), Ki67≥14% (OR=8.976, P=0.015), larger lesion size (OR=1.038, P=0.045), and higher Emean (OR=2.237, P=0.006) were observed to be the independent factors of NSLN metastasis. Based on the above four independent predictors, a nomogram was conducted to predict the risk of the NSLN metastasis in early-stage breast cancer patients with 1 or 2 positive SLNs. The nomogram showed good discrimination in the prediction of NSLN metastasis, with bias-corrected C-index of 0.855 (95% CI, 0.754-0.956) and 0.853 (95% CI, 0.724-0.983) in the training and validation cohorts, respectively. Furthermore, the AUC was 0.877 (95%CI: 0.776- 0.978) and 0.861 (95%CI: 0.732-0.991), respectively, indicating a good performance of the nomogram. The calibration curve suggested a satisfactory agreement between the predictive and actual risk in both the training (χ2 = 11.484, P=0.176, HL test) and validation (χ2 = 6.247, p = 0.620, HL test) cohorts, and the obvious clinical nets were revealed by DCA.

**Conclusions:**

We conducted a satisfactory nomogram model to evaluate the risk of NSLN metastasis in early-stage breast cancer patients with 1 or 2 SLN metastases. This model could be considered as an ancillary tool to help such patients to be selectively exempted from ALND.

## Introduction

Breast cancer, the primary factor of tumor-related morbidity in women worldwide, has grown rapidly in recent years, presenting a great threat to human health (1). According to the published data, breast cancer has become the leading cancer after surpassing lung cancer in the number of new cases per year, and is also a significant cause of tumor-related deaths (2). It is well known that axillary lymph node (ALN) status is a crucial factor in evaluating the staging, prognosis, and treatment of breast cancer. Sentinel lymph node biopsy (SLNB) can be used for accurately assessing the axillary lymph node status of patients with early-stage breast cancer and has been widely used in clinical practice (3). Patients with positive sentinel lymph node (SLN) unavoidably receive axillary lymph node dissection (ALND), despite postoperative complications and poorer quality of life (4). Unfortunately, several studies have reported that more than half of breast cancer patients with positive SLN who receive ALND do not have non-SLN (NSLN) metastasis, implying that ALND is unnecessary for all these patients (5–7), which have led to a debate on the need for routine ALND in early-stage breast cancer patients with 1 or 2 positive SLNs. The National Comprehensive Cancer Network guidelines advise that early-stage breast cancer patients with 1 or 2 positive SLNs who receive breast-conserving surgery and radiotherapy are exempt from ALND. Meanwhile, the nomograms were developed by Kimberly Van Zee (8), Tenon (9), and Helsinki University (10) for the prediction of the probability of NSLN metastasis in patients with early-stage breast cancer who had SLN metastasis, which are all conducted in developed countries. However, few nomogram models have been conducted in developing countries represented by China. The rate of breast-conserving surgery is very low in China, compared with that in developed countries; most breast cancer patients receive mastectomy (11). It is great important to explore the possibility of omitting ALND in early-stage breast cancer patients with 1 or 2 positive SLNs in China.

The stiffness of malignant lesion is greater compared with the benign lesion, because tumor stiffness is associated with the degree of tumor malignancy (12). Ultrasound elastography has been reported to be able to quantitatively and qualitatively measure tumor stiffness, which is important for distinguishing benign or malignant lesion (13, 14). Recent studies showed the use of ultrasound elastography in the examination of many organs, such as lymph nodes, liver, and thyroid (15, 16). Shear wave elastography (SWE), a novel elastography technique, provides quantitative information on measuring tissue stiffness (17, 18). Recently, SWE was reported to be associated with the axillary lymph node metastasis in breast cancer (19). However, no nomogram based on SWE was developed for the prediction of the axillary lymph node metastasis in breast cancer.

This study aimed to develop a nomogram based on elastography for the prediction of the risk of NSLN metastasis in early-stage breast cancer patients with 1 or 2 positive SLNs.

## Patients and methods

### Patients

Clinical data of early-stage breast cancer patients diagnosed in the Hulunbuir People’s Hospital and Wuxi Huishan District People’s Hospital from October 2019 to November 2022 were retrospectively collected. Inclusion criteria: (1) 18 years of age or older; (2) 1 or 2 SLN metastases; (3) successful SLNB and ALND; (4) first-time diagnosis of breast cancer; (5) receiving mastectomy. Exclusion criteria: (1) Treated with neoadjuvant chemotherapy; (2) Presence of other tumors; (3) Stage IV breast cancer patients; (4) Missing clinical data. A total of 118 early-stage breast cancer patients with 1 or 2 positive SLNs were eventually enrolled in this study. In addition, those patients enrolled from Hulunbuir People’s Hospital were assigned to the training cohort (n=82), and those enrolled from Wuxi Huishan District People’s Hospital were assigned to the validation cohort (n=36).

The Ethics Committee of the Hulunbuir People’s Hospital (No. 2021SYY-021) and Wuxi Huishan District People’s Hospital (No. HYLL20220510001) approved this study. Informed consents were provided by participants.

### Data collection

The clinical data of enrolled patients were collected, including ultrasound characteristics (Tumor size, Tumor shape, Tumor margin, Inner echo, Multifocality, Calcification, color doppler flow imaging [CDFI], and mean stiffness [Emean]) and clinicopathologic characteristics (age, body mass index [BMI], location of primary tumor, menstrual status, estrogen receptor, progesterone receptor, carbohydrate antigen [CA] 153, carcinoembryonic antigen [CEA], CA125, the number of negative SLNs, HER2 status, histological grade, and the Ki67 index).

### Construction of the nomogram

In the training cohort, the independent predictors were screened by logistic regression analysis and then were used to conducted the nomogram for the prediction of NSLN metastasis in early-stage breast cancer patients with 1 or 2 positive SLNs.

### Validation of the nomogram

The calibration curves and concordance index (C-index) were used to internally and externally assess the calibration and discrimination of the nomogram, respectively. Furthermore, the area under the receiver operating characteristic (ROC) curve (AUC) was calculated to explore the performance of the nomogram, and Hosmer-Lemeshow (HL) test was applied to the assessment of the calibration of model and an insignificant test statistic represents a perfect calibration. The clinical usefulness of the model was determined by decision curve analysis (DCA).

### Statistical analysis

Mann-Whitney U test and chi-square test were used to compare the continuous data that were skewed distributed (age, BMI, tumor size, and Emean) and categorical data (location of primary tumor, menstrual status, estrogen receptor, progesterone receptor, CA153, CEA, CA125, the number of negative SLNs, HER2 status, histological grade, Ki67 index, tumor shape, tumor margin, inner echo, multifocality, CDFI), respectively. Following the univariate logistic regression, multivariable analysis was performed to find the independent predictors, which was the basis of the nomogram. The statistical analyses were conducted with Medcalc software (version 18.0, Ostend, Belgium) and R package version 3.7.

## Results

A total of 601 breast cancer patients were initially recruited. According to the inclusion and exclusion criteria, 118 early-stage breast cancer patients with 1 or 2 positive SLNs were finally enrolled and were assigned to the training cohort (n=82) and the validation cohort (n=36), respectively. No significant differences were observed in baseline characteristics, including clinicopathologic and ultrasound characteristics, between the training and validation cohorts (all P>0.05) (**Table 1**).

**Table 1:** Baseline characteristics of patients in the training and validation cohorts.

Variables	Training Cohort (n=82)	Validation Cohort (n=36)	*P* value
Clinicopathologic characteristics			
Age, years, median (IQR)	56.5 (38.3, 74.8)	53.0 (47.0, 63.5)	0.583^*^
BMI, kg/m^2^, median (IQR)	20.4 (19.3, 22.6)	21.0 (19.6, 22.6)	0.710^*^
Location of primary tumor, n (%)			0.773^#^
Outer upper quadrant	45 (54.9)	18 (50.0)	
Other quadrant	37 (45.1)	18 (50.0)	
Menstrual status, n (%)			1.000^#^
Premenopausal	42 (51.2)	19 (52.8)	
Postmenopausal	40 (48.8)	17 (47.2)	
Estrogen receptor, n (%)			0.985^#^
Positive	61 (74.4)	26 (72.2)	
Negative	21 (25.6)	10 (27.8)	
Progesterone receptor, n (%)			0.847^#^
Positive	60 (73.2)	25 (69.4)	
Negative	22 (26.8)	11 (30.6)	
CA153, n (%)			1.000^#^
Positive	19 (23.2)	9 (25.0)	
Negative	63 (76.8)	27 (75.0)	
CEA, n (%)			0.887^#^
Positive	9 (11.0)	5 (13.9)	
Negative	73 (89.0)	31 (86.1)	
CA125, n (%)			1.000^#^
Positive	10 (12.2)	4 (11.1)	
Negative	72 (87.8)	32 (88.9)	
Negative SLNs, n (%)			0.997^#^
0–2	29 (35.4)	12 (33.3)	
>2	53 (64.6)	24 (66.7)	
HER2, n (%)			1.000^#^
Positive	20 (24.4)	9 (25.0)	
Negative	62 (75.6)	27 (75.0)	
Histological grade, n (%)			0.583^#^
I	16 (19.5)	7 (19.4)	
II	48 (58.5)	18 (50.0)	
III	18 (22.0)	11 (30.6)	
Ki67, n (%)			1.000^#^
<14%	26 (31.7)	11 (30.6)	
≥14%	56 (68.3)	25 (69.4)	
Ultrasound characteristics			
Tumor size, mm, median (IQR)	16.5 (11.0, 24.)	20.50 (11.0, 36.3)	0.243^*^
Tumor shape, n (%)			1.000^#^
Regular	6 (7.3)	2 (5.6)	
Irregular	76 (92.7)	34 (94.4)	
Tumor margin, n (%)			1.000^#^
Distinct	6 (7.3)	2 (5.6)	
Indistinct	76 (92.7)	34 (94.4)	
Inner echo, n (%)			0.747^#^
Even	6 (7.3)	4 (11.1)	
Uneven	76 (92.7)	32 (88.9)	
Multifocality, n (%)			1.000^#^
Yes	15 (18.3)	7 (19.4)	
No	67 (81.7)	29 (80.6)	
Calcification, n (%)			0.965^#^
Present	39 (47.6)	18 (50.0)	
Absent	43 (52.4)	18 (50.0)	
CDFI, n (%)			0.788^#^
0	0	0	
1	12 (14.6)	7 (19.4)	
2	66 (80.5)	27 (75.0)	
3	4 (4.9)	2 (5.6)	
Emean, kPa, median (IQR)	42.2 (33.6, 55.6)	44.2 (33.7, 55.8)	0.986^*^

^*^, for chi-square test; ^#^, for Mann-Whitney U-test. IQR, inter-quartile range; BMI, body mass index; CA, carbohydrate Antigen; CEA, carcinoembryonic antigen; SLN, sentinel lymph node; HER2, human epidermal growth factor receptor 2; CDFI: color doppler ﬂow imaging; Emean, mean stiffness.

### Independent predictors for preterm delivery

**Figure 1:**
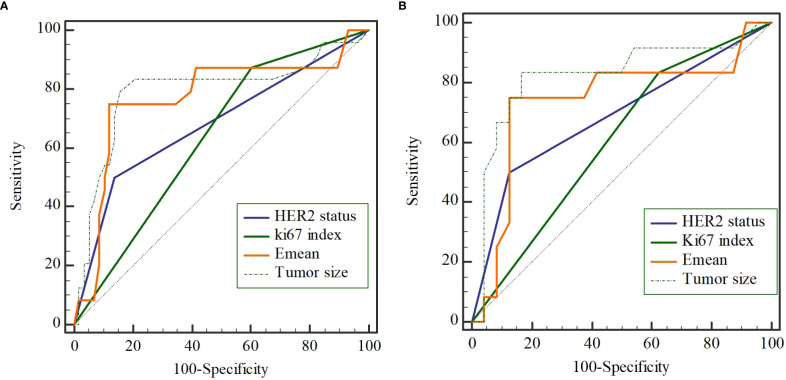
The performance of 4 independent predictors on non-sentinel lymph node (NSLN) metastasis verified by the receiver operating characteristic (ROC) curve in the training cohort **(A)** and the validation cohort **(B)**.

The univariate analysis based on clinicopathologic and ultrasound characteristics were performed to explore the potential predictors of NSLN metastasis in the training cohort. As shown in **Table 2**, only HER2 status (*P*=0.001), Ki67 index (*P*=0.023), tumor size (*P*=0.002), and Emean (*P*<0.001) were detected to be significantly associated with NSLN metastasis, which was confirmed in **Supplementary Table 1**. Furthermore, multivariable analysis showed that the enrolled patients with positive HER2 expression (OR=6.179, *P*=0.013), Ki67≥14% (OR=8.976, *P*=0.015), larger lesion size (OR=1.038, *P*=0.045), and higher Emean (OR=2.237, *P*=0.006) were observed to be the independent factors of NSLN metastasis (**Table 2**). To further observe the performance of the above independent factors for NSLN metastasis, we applied ROC curve. As shown in **Figures 1A, B**, the AUC of HER2 status, Ki67 index, tumor size, and Emean in the training cohort were 0.681, 0.636, 0.778, and 0.770, respectively, and the AUC in the validation cohort were 0.688, 0.604, 0.753, and 0.740, respectively, which further verified the performance of these four independent factors for NSLN metastasis.

**Table 2:** Univariate and multivariate logistic analyses to determine the independent predictors associated with NSLN metastasis in the training cohort.

Variables	Univariate logistic regression			Multivariate logistic regression		
**Clinicopathologic characteristics**	*P* value	OR	95% CI	*P* value	OR	95% CI
Age (years)	0.113	1.021	0.996-1.049			
BMI (kg/m^2^)	0.947	1.005	0.850-1.177			
Location of primary tumor (Outer upper quadrant vs. Other quadrant)	0.686	1.219	0.468-3.254			
Menstrual status, (Premenopausal vs. Postmenopausal)	0.531	0.737	0.280-1.913			
Estrogen receptor (Positive vs. Negative)	0.525	1.448	0.485-4.942			
Progesterone receptor (Positive vs.Negative)	0.759	0.847	0.299-2.547			
CA153 (Positive vs. Negative)	0.747	0.827	0.240-2.513			
CEA (Positive vs. Negative)	0.232	0.272	0.014-1.609			
CA125 (Positive vs. Negative)	0.957	1.041	0.209-4.146			
Negative SLNs (>2 vs. 0–2)	0.451	0.674	0.229-1.834			
HER2 (Positive vs. Negative)	0.001	6.250	2.139-19.449	0.013	6.179	1.554-28.697
Histological grade						
II vs. I	0.748	1.235	0.358-5.008			
III vs. I	0.596	1.500	0.340-7.185			
Ki67 (≥14% vs. <14%)	0.023	4.600	1.383-21.056	0.015	8.976	1.824-65.832
Ultrasound characteristics						
Tumor size (mm)	0.002	1.057	1.025-1.101	0.045	1.038	1.004-1.083
Tumor shape (Regular vs. Irregular)	0.491	0.461	0.023-3.071			
Tumor margin (Indistinct vs. Distinct)	0.490	2.170	0.326-42.805			
Inner echo (Uneven vs. Even)	0.820	0.815	0.148-6.181			
Multifocality (Yes vs. No)	0.388	0.548	0.116-1.946			
Calcification (Present vs. Absent)	0.493	0.714	0.268-1.856			
CDFI						
II vs. I	0.712	1.304	0.346-6.340			
III vs. I	1.000	1.000	0.041-12.184			
Emean, kPa, median (IQR)	<0.001	2.542	1.601-4.310	0.006	2.237	1.293-4.124

NSLN, non-sentinel lymph node; BMI, body mass index; CA, carbohydrate Antigen; CEA, carcinoembryonic antigen; HER2, human epidermal growth factor receptor 2; CDFI: color doppler ﬂow imaging; Emean, mean stiffness; IQR, inter-quartile range; OR, odds ratio; CI, confidence interval.

### Development and validation of the nomogram

**Figure 2:**
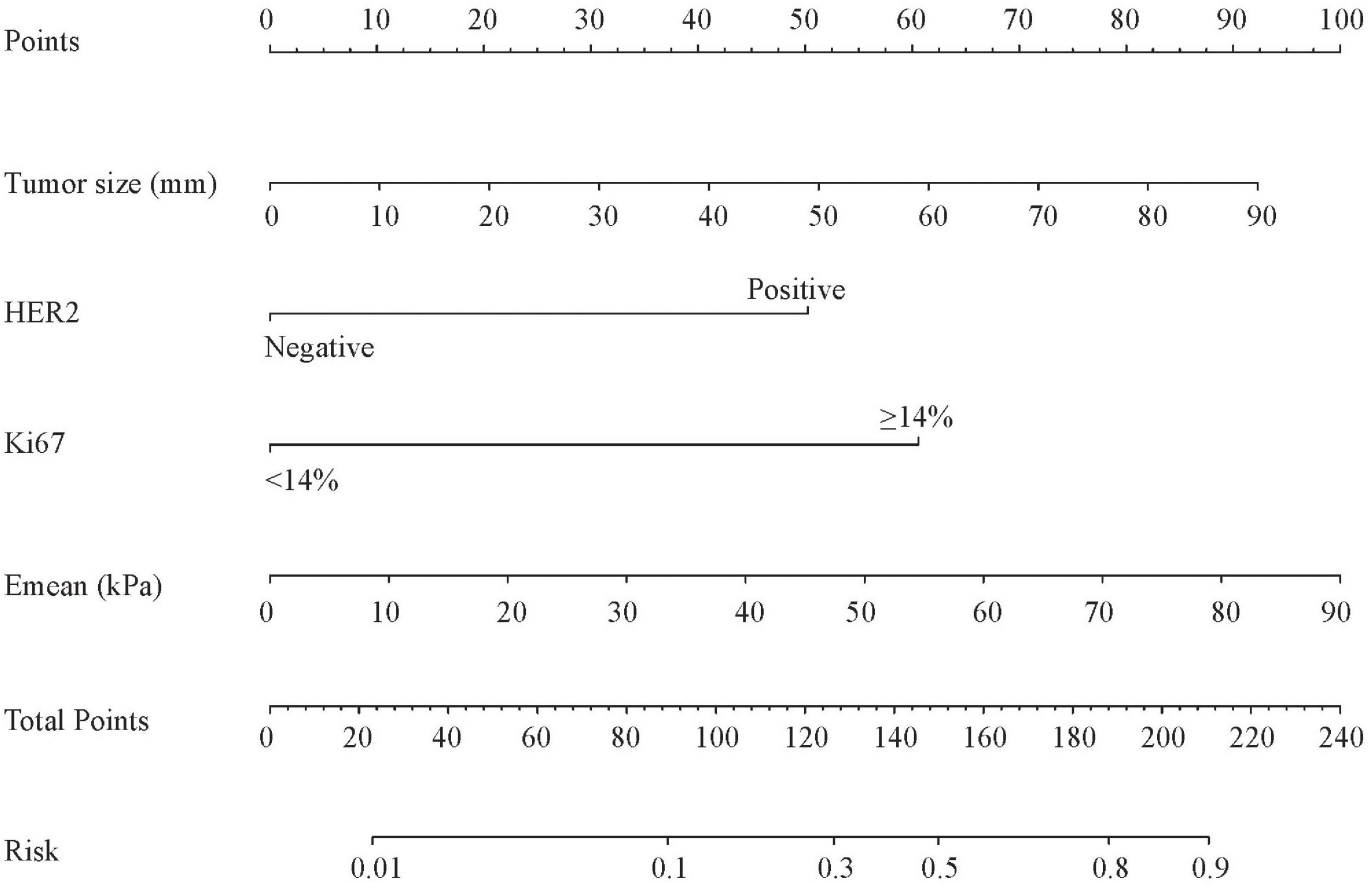
The nomogram predicting the risk of non-sentinel lymph node metastasis in breast cancer patients with 1 or 2 sentinel lymph node metastases. HER2, human epidermal growth factor receptor 2; Emean, mean stiffness.

**Figure 3:**
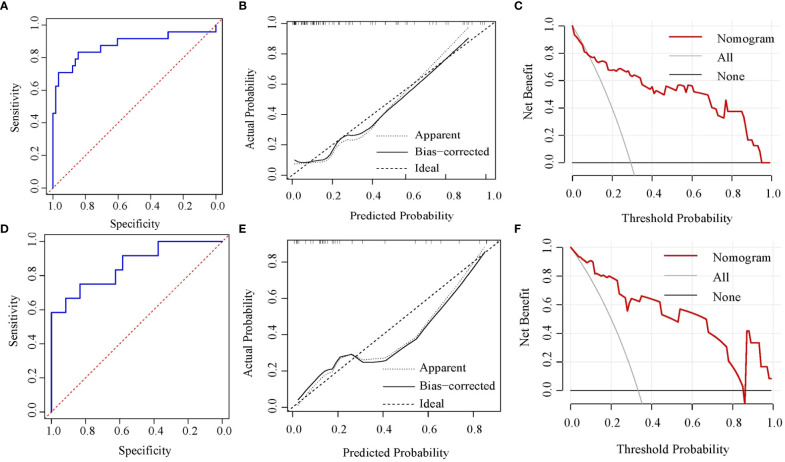
Nomogram verification [**(A-C)** for training cohort and **(D-F)** for validation cohort]. **(A, D)** ROC curves; **(B, E)** calibration plots; **(C, F)** DCA plots. ROC, receiver operating characteristic; DCA, decision curve analysis.

Based on the above four independent predictors, a nomogram was conducted to predict the risk of the NSLN metastasis in early-stage breast cancer patients with 1 or 2 positive SLNs. As shown in **Figure 2**, Emean had the greatest impact on the NSLN metastasis, with a maximum score of 100. The nomogram showed good discrimination in the prediction of NSLN metastasis, with bias-corrected C-index of 0.855 (95% CI, 0.754-0.956) and 0.853 (95% CI, 0.724-0.983) in the training and validation cohorts, respectively. Furthermore, as shown in **Figures 3A, D**, the AUC was 0.877 (95%CI: 0.776- 0.978) and 0.861 (95%CI: 0.732-0.991), respectively, indicating a good performance of the nomogram. The calibration curve suggested a satisfactory agreement between the predictive and actual risk in both the training (χ^2 =^ 11.484, *P*=0.176, HL test) and validation (χ^2 =^ 6.247, p = 0.620, HL test) cohorts (**Figure 3B, E**), and the obvious clinical nets were revealed by DCA (**Figure 3C, F**).

### Utility of the nomogram

The nomogram can be used to calculate the scores of each independent predictor, and the predicted probability that corresponds to the total points calculated by the sum of the scores of each independent predictor was the risk of NSLN metastasis in early-stage breast cancer patients with 1 or 2 positive SLNs. For example, when a patient with 50mm in lesion size, positive HER-2 expression, Ki67<14%, and 60kPa in Emean, the total scores were about 169. It showed that the risk of a patient with NSLN metastasis was about 60%.

## Discussion

The routine ALND therapy for breast cancer patients with 1 or 2 SLN metastases is overtreatment because there is no further NSLN metastasis in approximately 60% of such patients, while positive lymph nodes other than SLN, if not removed, can cause residual tumor, leading to axillary recurrence (5–7). Thus, the development of a model that accurately predicts NSLN metastasis could improve the management of patients with positive SLNs. Medical nomograms graphically present statistical prediction models using biological and clinical variables to give the risk of a clinical event, such as death or lymph node metastasis, for a given individual (20). Preoperative nomograms can be used to assess the probability of lymph node metastases, helping clinicians find patients who may benefit more from more extensive surgery (21, 22). Our retrospective study aimed to develop a nomogram based on elastography for the prediction of the risk of NSLN metastasis in early-stage breast cancer patients with 1 or 2 positive SLNs. A prediction model was conducted after we discovered the independent risk factors of NSLN metastasis. This satisfactory model was then verified using data from the training and validation cohorts, respectively.

As the tumor grows, it tends to become stiffer due to an increase in the fibrous component and a decrease in the necrotic component, which is well understood (23). SWE is a promising elastic imaging technology, allowing quantitative measurement of tissue stiffness (24). The lymph node metastasis of breast cancer has been reported to be associated with elastic modulus of breast masses (25). Moreover, the increased tumor stiffness is thought to be an easier infiltration of tumor cells into the tissue interstices, which is an independent predictor for the prediction of tumor recurrence (26–28). In this study, we found that the higher Emean was associated with a higher risk of NSLN metastasis, which may be interpreted by the histopathologic composition of the tumor. In addition, we visualized the effect of Emean on NSLN metastasis through the nomogram model.

The present study showed that tumor size and HER2 status were the independent risk factors of NSLN metastasis and that larger tumor size and positive HER2 expression leaded to a higher risk of NSLN metastasis. Other studies have also reported that these two factors are associated with NSLN metastasis (10, 29). In addition, we also found that Ki67 was the independent risk factor of NSLN metastasis, which was consistent with the conclusion that the high expression of Ki67 significantly improves the probability of lymph node metastasis (30).

The main strength of this study is the development of the nomogram with good performance for the prediction of the risk of NSLN metastasis in early-stage breast cancer patients with 1 or 2 positive SLNs in China. The risk of NSLN metastasis could be calculated repeatedly depending on the variable clinical status, and the increased risk of NSLN metastasis is dynamically evaluated, which would be very helpful for clinicians to adopt individualized treatment strategies. The C-index of the nomogram conducted by Liu et al. was 0.740 and 0.689 in the training and validation cohorts, respectively (31). Compared to previous results, our nomogram including elastography with greater C-index showed a better performance.

Two limitations exist in this study. First, due to the retrospective design of this study, clinical data for some patients were partially missing. Second, the patients enrolled in this study were from only 2 hospitals, which leaded to the nomogram conducted in this study was not confirmed in other populations. The research with a larger sample size will be performed to verify this nomogram model.

## Conclusions

We conducted a satisfactory nomogram model to evaluate the risk of NSLN metastasis in early-stage breast cancer patients with 1 or 2 SLN metastases. This model could be considered as an ancillary tool to help such patients to be selectively exempted from ALND.

## Data availability statement

The original contributions presented in the study are included in the article/**Supplementary Material**. Further inquiries can be directed to the corresponding authors.

## Ethics statement

The studies involving human participants were reviewed and approved by Hulunbuir People’s Hospital (No. 2021SYY-021) and Wuxi Huishan District People’s Hospital (No. HYLL20220510001). The patients/participants provided their written informed consent to participate in this study.

## Author contributions

Study design: HD, XZ, WW. Data collection and analysis: HD, JZ, GZ, XZ, WW. Supervision: HD, XZ, WW. Statistics: HD, JZ, GZ. Manuscript writing: HD. Manuscript revision: XZ, WW. Approval of the manuscript: all authors.

## Supplementary material

Supplementary Table 1 Baseline characteristics of patients between the term delivery and preterm delivery group in the training cohort.
