# Supplementary material for: An improved nomogram including elastography for the prediction of non-sentinel lymph node metastasis in breast cancer patients with 1 or 2 sentinel lymph node metastases
Source: Front Oncol. 2023 Jun 5;13:1196592. doi: 10.3389/fonc.2023.1196592 (PMC10277680; doi:10.3389/fonc.2023.1196592)
Supplement: Supplementary Table 1 — Baseline characteristics of patients between the term delivery and preterm delivery group in the training cohort. [file Table_1.docx]

Supplementary Table 1 Baseline characteristics of patients between the term delivery and preterm delivery group in the training cohort

| Variables | Non-NSLN metastasis (n=58) | NSLN metastasis (n=24) | *P* value |
| --- | --- | --- | --- |
| **Clinicopathologic characteristics** |  |  |  |
| Age, years, median (IQR) | 54.0 (35.3, 71.8) | 64.0 (46.8, 77.0) | 0.113^*^ |
| BMI, kg/m^2^, median (IQR) | 20.4 (19.1, 22.6) | 20.5 (19.4, 22.8) | 0.650^*^ |
| Location of primary tumor, n (%) |  |  | 0.872^#^ |
| Outer upper quadrant | 31 (53.4) | 14 (58.3) |  |
| Other quadrant | 27 (46.6) | 10 (41.7) |  |
| Menstrual status, n (%) |  |  | 0.700^#^ |
| Premenopausal | 31 (53.4) | 11 (45.8) |  |
| Postmenopausal | 27 (46.6) | 13 (51.2) |  |
| Estrogen receptor, n (%) |  |  | 0.719^#^ |
| Positive | 42 (72.4) | 19 (79.2) |  |
| Negative | 16 (27.6) | 5 (20.8) |  |
| Progesterone receptor, n (%) |  |  | 0.973^#^ |
| Positive | 43 (74.1) | 17 (70.8) |  |
| Negative | 15 (25.9) | 7 (29.2) |  |
| CA153, n (%) |  |  | 0.972^#^ |
| Positive | 14 (24.1) | 5 (20.8) |  |
| Negative | 44 (75.9) | 19 (79.2) |  |
| CEA, n (%) |  |  | 0.379^#^ |
| Positive | 8 (13.8) | 1 (4.2) |  |
| Negative | 50 (86.2) | 23 (95.8) |  |
| CA125, n (%) |  |  | 1.000^#^ |
| Positive | 7 (12.1) | 3 (12.5) |  |
| Negative | 51 (87.9) | 21 (87.5) |  |
| Negative SLNs, n (%) |  |  | 0.616^#^ |
| 0–2 | 22 (37.9) | 7 (29.2) |  |
| >2 | 36 (62.1) | 17 (70.8) |  |
| HER2, n (%) |  |  | 0.001^#^ |
| Positive | 8 (13.8) | 12 (50.0) |  |
| Negative | 50 (86.2) | 12 (50.0) |  |
| Histological grade, n (%) |  |  | 0.867^#^ |
| I | 12 (20.7) | 4 (16.7) |  |
| II | 34 (58.6) | 14 (58.3) |  |
| III | 12 (20.7) | 6 (25.0) |  |
| Ki67, n (%) |  |  | 0.032^#^ |
| <14 | 23 (39.7) | 3 (12.5) |  |
| ≥14 | 35 (60.3) | 21 (87.5) |  |
| **Ultrasound characteristics** |  |  |  |
| Tumor size, mm, median (IQR) | 14.0 (11.0, 18.8) | 27.5 (22.0, 40.0) | <0.001^*^ |
| Tumor shape, n (%) |  |  | 0.811^#^ |
| Regular | 5 (8.6) | 1 (4.2) |  |
| Irregular | 53 (91.4) | 23 (95.8) |  |
| Tumor margin, n (%) |  |  | 0.811^#^ |
| Distinct | 5 (8.6) | 1 (4.2) |  |
| Indistinct | 53 (91.4) | 23 (95.8) |  |
| Inner echo, n (%) |  |  | 1.000^#^ |
| Even | 4 (6.9) | 2 (8.3) |  |
| Uneven | 54 (93.1) | 22 (91.7) |  |
| Multifocality, n (%) |  |  | 0.576^#^ |
| Yes | 12 (20.7) | 3 (12.5) |  |
| No | 46 (79.3) | 21 (87.5) |  |
| Calcification, n (%) |  |  | 0.657^#^ |
| Present | 29 (50.0) | 10 (41.7) |  |
| Absent | 29 (50.0) | 14 (58.3) |  |
| CDFI, n (%) |  |  | 0.916^#^ |
| 0 | 0 | 0 |  |
| 1 | 9 (15.5) | 3 (12.5) |  |
| 2 | 46 (79.3) | 20 (83.3) |  |
| 3 | 3 (5.2) | 1 (4.2) |  |
| Emean, kPa, median (IQR) | 35.0 (20.6, 55.6) | 49.7 (35.0, 60.9) | <0.001^*^ |

*, for chi-square test; #, for Mann-Whitney U-test. IQR, inter-quartile range; BMI, body mass index; CA, carbohydrate Antigen; CEA, carcinoembryonic antigen; SLN, sentinel lymph node; HER2, human epidermal growth factor receptor 2; CDFI: color doppler ﬂow imaging; Emean, mean stiffness; NSLN, non-sentinel lymph node.
